# The Influence of Closeness Centrality on Lexical Processing

**DOI:** 10.3389/fpsyg.2017.01683

**Published:** 2017-09-26

**Authors:** Rutherford Goldstein, Michael S. Vitevitch

**Affiliations:** Department of Psychology, University of Kansas, Lawrence, KS, United States

**Keywords:** network science, lexical search, spoken word recognition, closeness centrality

## Abstract

The present study examined how the network science measure known as closeness centrality (which measures the average distance between a node and all other nodes in the network) influences lexical processing. In the mental lexicon, a word such as CAN has high closeness centrality, because it is close to many other words in the lexicon. Whereas, a word such as CURE has low closeness centrality because it is far from other words in the lexicon. In an auditory lexical decision task (Experiment 1) participants responded more quickly to words with high closeness centrality. In Experiment 2 an auditory lexical decision task was again used, but with a wider range of stimulus characteristics. Although, there was no main effect of closeness centrality in Experiment 2, an interaction between closeness centrality and frequency of occurrence was observed on reaction times. The results are explained in terms of partial activation gradually strengthening over time word-forms that are centrally located in the phonological network.

## Introduction

**Figure 1:**
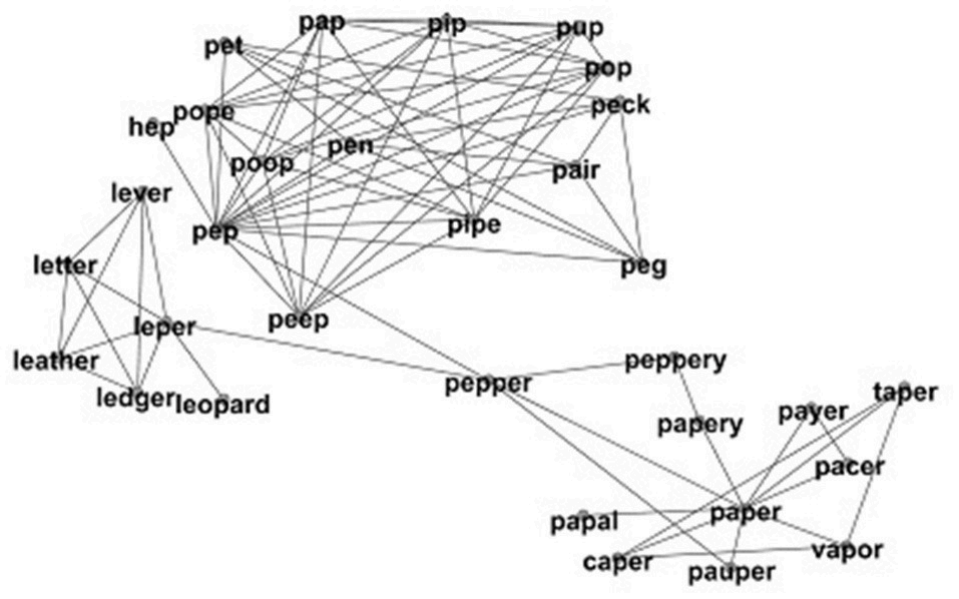
A portion of phonological network showing the word PEPPER, the neighbors of the word PEPPER, and the neighbors of those neighbors. A link is placed between words when they are phonological neighbors of each other. Adapted from Vitevitch (2008).

Complex networks are increasingly being used to better understand various aspects of human cognition (e.g., Steyvers and Tenenbaum, 2005; Hills et al., 2009). A network is constructed out of nodes (representing entities) and links (representing relationships between those entities). In the present study nodes represent phonological word-forms in that part of memory known as the mental lexicon, and links between nodes indicate that the words are phonologically similar to each other (as in the network created in Vitevitch, 2008). See Figure 1 for a subset of this network.

**Figure 2:**
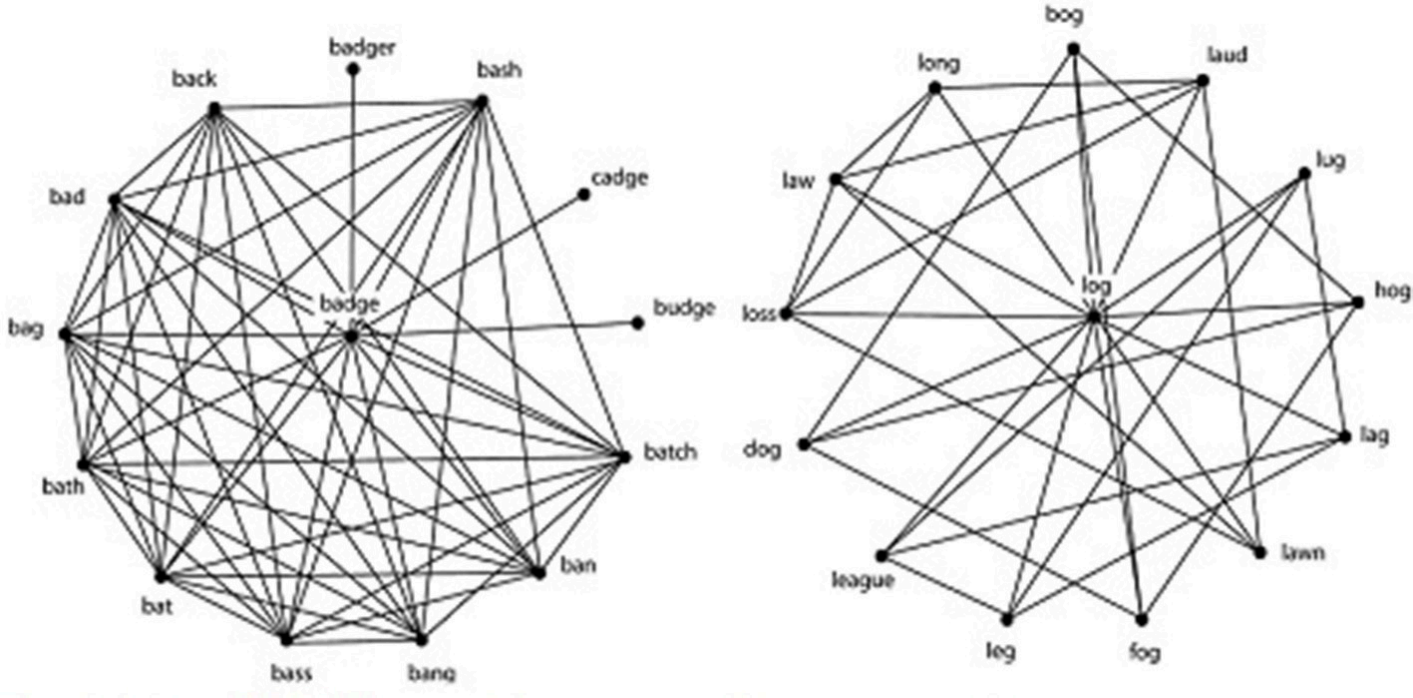
The word BADGE on the left has many neighbors that are neighbors of each other and therefore has a high C. The word LOG on the right has few neighbors that are neighbors of each other and therefore has a low C. Notice that both words have the same number of phonological neighbors: 13. Used with the permission of the author: (Chan and Vitevitch, 2009).

A central tenet of network science is that the structure of the network influences the processes that operate in that network (Watts and Strogatz, 1998). One measure of the structure of the network is called the clustering coefficient, or *C*, which measures how many nodes connected to a target node are also connected to each other. In the phonological lexicon, *C* is a measure of the extent to which phonological neighbors of a word are also phonological neighbors of each other. For example, the word BADGE has the neighbors BAG, BAD, and BAT, which are also neighbors of each other. A value of *C* = 1 indicates that all the neighbors of a word are neighbors of each other, whereas a value of *C* = 0 indicates that no neighbors of a word are neighbors of each other. As illustrated in Figure 2, BADGE has a high *C* value, because many of the neighbors of BADGE are also neighbors of each other. In contrast, the word LOG has a low *C* value because its neighbors tend to not be neighbors of each other.

The clustering coefficient of a word has been shown to influence a number of language- and memory-related processes. Chan and Vitevitch (2009) found that spoken word recognition was influenced by *C* in both a perceptual identification task and a lexical decision task. A processing advantage (i.e., higher accuracy rates or faster reaction times) was observed for low *C* words in both tasks (see Vitevitch et al., 2011 for a computer simulation of the results). Chan and Vitevitch (2010) also explored how speech production was influenced by *C*. The results of a speech error corpus analysis and a picture naming task show that high *C* words were produced with greater errors and with slower reaction times compared to low *C* words. However, in the case of word-learning, Goldstein and Vitevitch (2014) found that novel words with high *C* were better learned than novel words with low *C* words due to the reverberating activation strengthening the nascent representations of novel words with high *C*. See Vitevitch et al. (2012) for the influence of *C* on short-term and long-term memory. The findings from these studies of the clustering coefficient demonstrate that the structure of the network *immediately surrounding* a single word influences various memory- and language-related processes.

The *overall* structure of the network has also been shown to influence language-related processing. Vitevitch and Goldstein (2014) identified “keyplayers” in the mental lexicon, or nodes whose removal fractures a connected network into smaller, disconnected components (Borgatti, 2006). In a perceptual identification task with degraded stimuli, a naming task, and a lexical decision task the “keywords” were responded to more quickly and accurately than another set of words that were matched on a number of relevant lexical characteristics. Thus, the structure of the network immediately surrounding a word as well as the structure further away from a word influences certain lexical processes (see Vitevitch and Castro, 2015 for a review of how several other network measures influence language processing).

In the present study we further examined how the overall structure of the network influences processing using the network measure known as *closeness centrality*. In the lexical network examined here closeness centrality measures the average number of links between a word and all other words in the lexicon. More precisely, see Equation 1, closeness centrality is the inverse of “farness,” or the average number of links that must be traversed from a node in the network to all other nodes in the network:

(1) $$\begin{array}{r} C_{v}=\;\frac{1}{\Sigma_{u\in V}d\left(v,u\right)} \end{array}$$

*d*(*v, u*) refers to the shortest path between nodes *v* and *u*. Σ refers to the sum of the path lengths from node *v* to all other nodes in the network (i.e., “farness”).

In Figure 1, the word PEPPER is centrally located in the surrounding network. In other words, PEPPER is relatively few links away from all other words in the network and would have a high closeness centrality in the subset of the phonological network portrayed in Figure 1. The word PAPAL is not centrally located in the network. PAPAL is relatively many links away (or far) from all other words in the network and would have a low closeness centrality in the subset of the phonological network portrayed in Figure 1.

Closeness centrality is a global network measure, it measures the relationship between a single word and all other words in the lexicon. However, it may help to explain closeness centrality in terms of a local network measure, such as neighborhood density. Neighborhood density in the lexicon is a measurement of the number of words that differ by a single phoneme. For example, the word PEPPER in Figure 1 has the five neighbors of PEP, LEPER, PEPPERY, PAUPER, and PAPER. Therefore, PEPPER has a neighborhood density value of five. Neighborhood density measures the number of words that are one network link away from a given word (i.e., a local network measure). Closeness centrality on the other hand measures the average number of network links from a given word and *all the other words* in the lexicon (i.e., a global network measure). Local and global network measures assess different aspects of a network.

Closeness centrality ranges from 0 to 1. Values close to 0 indicate that a given node is “far” from other nodes in the network (i.e., many links must be traversed to get from that node to other nodes in the network), whereas a value close to 1 indicates that a given node is “close” to other nodes in the network (i.e., few links must be traversed to get from that node to other nodes in the network). For example, the word CAN is a small average number of links away from every other word in the lexicon and has a high closeness centrality (i.e., CAN is “close” to the rest of the lexicon). The word CURE is a large average number of links away from every other word in the lexicon and has a low closeness centrality (i.e., CURE is “far” from the rest of the lexicon).

Closeness centrality values vary depending on the size of the network (Freeman, 1979); larger networks will have more connections to be traversed and therefore tend to have “lower” centrality values than smaller networks. In order to remove the influence of network size on closeness centrality values some researchers use a normalized closeness centrality value (Freeman, 1979). However, in the present study we are not comparing closeness centrality values across multiple networks that vary in size, so we elected not to normalize the closeness centrality values. To give the reader some perspective the lexical network we examined (the same one used in Vitevitch, 2008 which contained ~6,500 words in the giant component) had a range of closeness centrality values from the lowest value of 0.0001 to the highest value of 0.08.

A pioneering study by Iyengar et al. (2012) suggests that closeness centrality may be important for lexical processing. Iyengar et al. developed a “word-morph” game in which participants were given a start word (e.g., BAD) and were instructed to form another real word by changing one letter at a time (e.g., BAD 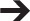 BAT is acceptable whereas BAD 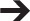 BAC is not acceptable) to navigate their way through the lexicon (e.g., BAD 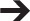 BAT 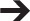 BIT 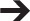 BUT) to form a specified “end” word (e.g., NUT). Participants soon discovered trying to take as direct a route as possible would often not lead to success, and instead discovered the utility of certain “landmark” words. Much like physical landmarks in spatial navigation, navigating from the start word to a landmark word in the lexicon made it easier to then navigate to the end word in the word-morph game. Upon further analysis, Iyengar et al. discovered that the landmark words were high in closeness centrality. Given the important role that words with high closeness centrality played in the word-morph game, we wondered how closeness centrality might influence other language-related processes.

Due to the proximity of words with high closeness centrality to all other words in the lexicon, one might reason that such words will have processing disadvantages (i.e., lower accuracy rates or slower reaction times) because of increased competition with other words in the lexicon as predicted by widely accepted models of spoken word recognition (e.g., McClelland and Elman, 1986; Norris, 1994; Luce and Pisoni, 1998). Alternatively, words with high closeness centrality may exhibit a processing advantage due to the increased amount of indirect partial activation they receive from many “distant” words in the lexicon. The consequences of a word being partially activated on a repeated basis is not entirely understood, but evidence from Vitevitch and Goldstein (2014) suggests that repeated partial activation of a word-form may be beneficial. Vitevitch and Goldstein observed that words in “key” positions in the lexicon, or that keep the network from breaking apart, may receive a large amount of partial activation, which over time strengthens those representations, possibly accounting for the processing advantages they observed for keywords.

A similar finding by Sommers and Lewis (1999) using the phonological false memory phenomenon (see Roediger and McDermott, 1995 for semantic false memories) also shows in a memory-related task that accrued partial activation may influence subsequent processing. Sommers and Lewis (1999) found that participants often falsely remembered lure-words that were not presented during the exposure phase of the experiment if many similar sounding words, or phonological neighbors, of the lure word were presented during the exposure phase. For example, the word SLEEP might be falsely remembered if the words LEAP, SEEP, and SHEEP were presented during study due to the nearby retrieved words LEAP, SEEP, and SHEEP partially and repeatedly activating the word SLEEP. Although phonological false memories are an example of erroneous retrieval it is reasonable to apply the same mechanism regarding repeated, partial activation to the present case of words with high closeness centrality (Similar ideas about accumulated activation leading to processing benefits can also be found in Node Structure Theory developed by MacKay, 1982). Due to the proximity of words with high closeness centrality to many other words we reasoned that they will receive a great deal of partial activation, and that this partial activation might over time strengthen those representations leading to a processing benefit for such words. We report the results of two experiments that examined the influence of closeness centrality on lexical processing.

## Experiment 1

To examine how closeness centrality influences language processing, specifically spoken-word recognition, a traditional task from psycholinguistics was used, the auditory lexical decision task. The auditory lexical decision task requires participants to respond to stimuli by making a “word” or “non-word” judgment. Examining the influence of closeness centrality in a conventional task that assesses certain aspects of lexical processing will help establish if closeness centrality influences spoken word recognition.

### Methods

#### Participants

All 48 participants in Experiment 1 were healthy, college-aged adults sampled from the University of Kansas community. All participants were right-handed native English speakers with normal hearing as assessed through self-report. Participants received partial course credit for their participation.

#### Materials

The stimuli used in Experiment 1 consisted of 40 monosyllabic words split into two groups that varied in closeness centrality: a high closeness centrality group and low closeness centrality group. The two groups of words were controlled on several variables that have been shown to influence lexical processing: frequency of occurrence as measured by Kucera and Francis (1967) [*F*_(1, 38)_ = 0.001, *p* < 0.99; High: *M* = 2.08, *SD* = 0.76; Low: *M* = 2.09, *SD* = 0.83], frequency of occurrence as measured by Brysbaert and New (2009) [*F*_(1, 38)_ = 0.02, *p* < 0.88; High: *M* = 73, *SD* = 193; Low: *M* = 83, *SD* = 228] segment probability [Vitevitch and Luce, 1998; *F*_(1, 38)_ = 0.71, *p* < 0.41; High: *M* = 0.04, *SD* = 0.009; Low: *M* = 0.04, *SD* = 0.005], biphone probability [Vitevitch and Luce, 1998; *F*_(1, 38)_ = 0.041, *p* < 0.84; High: *M* = 0.002, *SD* = 0.002; Low: *M* = 0.002, *SD* = 0.001], neighborhood density/degree [Luce and Pisoni, 1998; *F*_(1, 38)_ = 1.14, *p* < 0.29; High: *M* = 16.6, *SD* = 2.96; Low: *M* = 15.7, *SD* = 1.97], neighborhood frequency [*F*_(1, 38)_ = 1.61, *p* < 0.21; High: *M* = 134.3, *SD* = 159.3; Low: *M* = 81.2, *SD* = 98.7], and word familiarity [familiarity ratings based on a 1–7 scale from Nusbaum et al., 1984; *F*_(1, 38)_ = 2.91, *p* < 0.09; High: *M* = 6.9, *SD* = 0.12; Low: *M* = 6.8, *SD* = 0.29].

In addition, some measures from network science that have been shown to influence lexical processing were also controlled: clustering coefficient [Chan and Vitevitch, 2009; *F*_(1, 38)_ = 0.041, *p* < 0.84; High: *M* = 0.38, *SD* = 0.13; Low: *M* = 0.35, *SD* = 0.09], and none of the words chosen as stimuli were keywords (Vitevitch and Goldstein, 2014). However, our variable of interest, closeness centrality, differed between the two groups [*F*_(1, 38)_ = 208, *p* < 0.001; High: *M* = 0.072, *SD* = 0.001; Low: *M* = 0.067, *SD* = 0.001].

Additionally, the durations of the sound files were equivalent for the two groups of words: stimulus onset time, measured from the start of the sound file to the beginning of the stimulus [*F*_(1, 38)_ = 1.99, *p* < 0.17; High: *M* = 20 ms, *SD* = 9 ms; Low: *M* = 20 ms, *SD* = 9 ms], duration of the stimulus [*F*_(1, 38)_ = 3.48, *p* < 0.07; High: *M* = 520 ms, *SD* = 60 ms; Low: *M* = 560 ms, *SD* = 80 ms], stimulus offset time, measured from the end of the stimulus to the end of the sound file [*F*_(1, 38)_ = 2.19, *p* < 0.15; High: *M* = 20 ms, *SD* = 7 ms; Low: *M* = 20 ms, *SD* = 7 ms], and overall duration of the sound file [*F*_(1, 38)_ = 3.70, *p* < 0.07; High: *M* = 570 ms, *SD* = 60 ms; Low: *M* = 600 ms, *SD* = 80 ms].

The non-words used in the lexical decision task were created by changing the last phoneme of the real word stimuli to create phonotactically legal non-words. See Appendix A in Supplementary Material for a list of the real words and the non-words that were used in Experiment 1.

#### Procedure

After obtaining informed consent participants were seated in front of an iMac computer running PsyScope 1.2.2 (Cohen et al., 1993), which controlled the presentation of stimuli and the collection of responses. Participants heard one of the randomly selected stimulus items through Beyerdynamic DT 100 headphones set at a comfortable listening level. Each stimulus was presented only once. After presentation of the stimulus, participants decided if they heard a non-word or a word and pressed a response button to indicate their choice. Reaction times were measured from the onset of the stimulus to the onset of the button press. A short practice session was administered at the start of the experiment in order to familiarize participants with the task.

### Results

For Experiment 1 the dependent variables of interest were reaction times and accuracy rates. For each dependent variable a multilevel model was created. Items were used as the level 1 units and participants as the level 2 units. The level 1 predictor of interest was closeness centrality of the word. The multilevel modeling analyses were conducted using the statistical software R (R Development Core Team, 2013) with the package “lme4” (Bates et al., 2012).

A binomial distribution (“correct” or “incorrect”), was used in the model examining accuracy rates. Responses that were either too long (>1,800 ms) or too short (<300 ms) were removed from the analysis, resulting in ~2% of the responses being removed. The cutoffs of >1,800 ms and <300 ms were used to remove outliers. The cutoffs were determined because they remove responses >2.5 standard deviations from the mean. Responses beyond the cutoffs usually represent an accidental premature key press or a slight distraction that caused the participant to miss the stimulus (such as a sneeze or cough). Closeness centrality was added to the model with a random slope and random intercept. The analysis showed a non-significant positive coefficient (β = 43.02, *p* = 0.21), indicating that accuracy tended to increase as closeness centrality increased. That is, participants tended to be more accurate when responding to words with high closeness centrality (*M* = 89.73, *SD* = 6.77) than to words with low closeness centrality (*M* = 86.49, *SD* = 7.24), however the difference between the groups was not significant at the 0.05 level. It is not surprising that there was no difference between the high and low closeness centrality groups, since the auditory lexical decision task is typically not very sensitive to differences in accuracy rates.

A Gaussian distribution was used in the model examining reaction times (measured in milliseconds). Responses that were too long (>1,800 ms) or too short (<300 ms) were removed from the analysis, resulting in ~2% of the responses being removed. Only correct responses were included in the reaction time analysis. Closeness centrality was added to the model with a random slope and random intercept. The analysis showed a decrease in reaction times for the words with high closeness centrality (*M* = 909 ms, *SD* = 74 ms) compared to words with low closeness centrality (*M* = 950 ms, *SD* = 130 ms) with a negative coefficient (β = –14,447, *p* < 0.001), indicating that reaction times were faster for words with high closeness centrality.

A possible explanation for the significant difference in reaction times between the words is the difference of the stimulus durations for the words with high vs. low closeness centrality. Although the difference between the groups was not statistically significant, the difference in stimulus durations between the groups was 40 ms, approximately the same difference in reaction times observed between words with high vs. low closeness centrality. To examine the possible influence of stimulus duration on the observed effect a multilevel model was created with stimulus duration as a predictor. The results showed that stimulus duration was a significant predictor (β = 3.95, *p* < 0.001), but closeness centrality was still a significant predictor as well (β = −1.04, *p* < 0.01), indicating that closeness centrality still influenced reaction time in the present experiment.

### Discussion

The results from Experiment 1 showed that words with high closeness centrality were responded to more quickly and accurately than words with low closeness centrality, providing evidence that closeness centrality influences spoken word recognition. We suggest that the processing advantage observed for words with high closeness centrality may stem from the advantageous position they occupy in the lexical network, allowing for partial activation to accrue benefits over time. That is, even though those specific words may not be retrieved themselves, they are close to many words that are retrieved. The partial activation these words receive when other nearby words are retrieved strengthens the representation of these words over time, providing processing benefits when these words finally are retrieved. In the case of words with low closeness centrality, they are located in areas of the lexicon that receive much less partial activation and therefore less of a processing advantage is obtained when nearby words are retrieved. Again, the partial activation is thought to influence lexical retrieval over long periods of time (i.e., years of exposure to words). The observed processing benefits are expected after years of exposure to words, activation spreading across the lexicon, and that activation accumulating in high closeness centrality words. The short-term exposure to the experimental stimuli is not expected to be the main source of differences in processing found in the experiments. See MacKay (1982) for more information on how long-term activation can influence lexical retrieval.

Additionally, the results from Experiment 1 cannot be explained by currently accepted models of spoken word recognition (e.g., McClelland and Elman, 1986; Norris, 1994; Luce and Pisoni, 1998). Such models would predict that words with high closeness centrality would be subject to increased competition and therefore responded to less quickly and accurately than words with low closeness centrality, which have competitors that were “further away” in the lexicon. Furthermore, currently accepted models of spoken word recognition predict processing differences based on characteristics of individual words (e.g., frequency of occurrence, word-length, phonotactic probability), not on where the word is located in the mental lexicon/lexical network. Recall that these characteristics of individual words were matched in the stimuli used in the present experiment (which means that currently accepted models of spoken word recognition would again predict no difference between these two sets of words). To test the claim that currently accepted models of spoken word recognition would show no difference between the high and low closeness centrality word groups used in Experiment 1, we attempted to simulate this experiment using the jTRACE model. However, only two words (both from the high closeness centrality condition) from Experiment 1 were found in the jTRACE lexicons, so we were unable to carry out the desired simulation.

The results from Experiment 1 further support the growing body of evidence that suggests that the structure of the lexicon influences cognitive processes such as spoken word recognition. Not only does the structure of the lexicon immediately surrounding a word influence processing as the results of Chan and Vitevitch (2009, 2010) suggest, but, as the current results suggest, the global structure of the lexicon influences processing as well.

## Experiment 2

Although, the words used as stimuli in Experiment 1 were representative of the majority of words in the lexicon (i.e., we sampled words with closeness centrality values from the range of values that contained most of the words in the giant component) we wished to explore how a broader range of closeness centrality values might influence the process of spoken word recognition (Note that the difference between the highest and lowest closeness centrality values in Experiment 1 was 0.0084, whereas the difference between the highest and lowest closeness centrality values of the stimuli used in Experiment 2 was 0.0675). In order to sample a broader range of words varying in closeness centrality we used bisyllabic words (compared to the monosyllabic words used in Experiment 1), and allowed other commonly investigated lexical characteristics to vary as well (instead of matching words on those variables as we did in Experiment 1). Our use of multi-level modeling in the present analysis enabled us to assess the influence of these other lexical variables on processing.

### Methods

#### Participants

All 37 participants in Experiment 2 were healthy, college-aged adults sampled from the University of Kansas community. All participants were right-handed native English speakers with normal hearing as assessed through self-report. Participants received partial course credit for their participation.

#### Materials

The stimuli used in Experiment 2 consisted of 80 words that contained four phonemes and two syllables. The non-words used in Experiment 2 were created by changing the last phoneme of the real words into a phonotactically legal non-word. For a list of stimuli used in Experiment 2 and the associated variable values see Appendix B in Supplementary Material. Words were initially chosen at random, however to ensure a representative range of lexical characteristics individual words were pseudo-randomly replaced if they were outliers on any given lexical characteristic (i.e., >2 standard deviations from the mean).

#### Procedure

After obtaining informed consent, the same procedure used in Experiment 1 was followed in the present experiment.

### Results

Separate series of multilevel models were created to assess the dependent variables of reaction times and accuracy rates using stepwise regression with all item-level (level 1) variables as fixed effects (participants as level 2 units). Item level variables were added as fixed effects due to the large range of variable values included in the stimuli. Level 1 predictors included closeness centrality, clustering coefficient, frequency of occurrence (Brysbaert and New, 2009), number of phonological neighbors/degree (Luce and Pisoni, 1998), segment probability (Vitevitch and Luce, 1998), biphone probability (Vitevitch and Luce, 1998), and neighborhood frequency (log transformed). See Table 1 for descriptive statistics of predictor variables. See Table 2 for correlation values between predictors. Non-word responses were not analyzed, and only responses between 300 and 1,800 ms were included in the analyses (1.5% of the data were dropped as outliers).

**Table 1:** Descriptive statistics of predictor variables used in Experiment 2.

	***C***	**Closeness centrality**	**Segment sum**	**Biphone sum**	**Neighborhood density**	**Neighborhood frequency**	**Brysbaert & new frequency**
Range	1	0.067	0.22	0.04	19	672	875
Mean	0.25	0.046	0.17	0.01	4.4	35	29
Standard deviation	0.29	0.02	0.05	0.007	3.7	86	116

**Table 2:** Correlation values between predictor variables used in Experiment 2.

	**Intercept**	**Closeness centrality**	**Brysbaert & new frequency**	***C***	**Neighborhood density**	**Segment sum**	**biphone sum**
Closeness centrality	−0.12						
Brysbaert & new frequency	−0.3	0.17					
*C*	−0.3	−0.14	0.11				
Neighborhood density	0.08	−0.3	−0.13	−0.04			
Segment sum	−0.71	−0.43	0.1	0.2	−0.09		
Biphone sum	0.22	0.14	0.01	−0.23	−0.35	−0.46	
Neighborhood frequency	−0.16	−0.2	−0.004	0.18	0.01	0.15	−0.09

For reaction times, a model was created without any interactions between predictors. The model was created using stepwise regression with all item-level (level 1) variables as fixed effects (participants as level 2 units). Predictor variables were not centered. This model indicated that several variables significantly predicted reaction times: frequency of occurrence, clustering coefficient, and log mean frequency of neighborhood. For frequency of occurrence, words that occurred in the language more often were responded to more quickly than words that occurred in the language less often (β = –2.22, *p* < 0.01), replicating the well-known and often observed influence of word-frequency on processing (Forster and Chambers, 1973). For the clustering coefficient (i.e., how many phonological neighbors of a word are also neighbors of each other), words with high clustering coefficient were responded to more slowly than words with low clustering coefficient (β = 37.82, *p* < 0.0001), replicating the result reported in Chan and Vitevitch (2009). No significant effect of closeness centrality on reaction times was observed.

Stimulus duration was shown to be a significant predictor in Experiment 1. Therefore, stimulus duration was added as a fixed effect in a model identical to the one described above. Stimulus duration was not a significant predictor (β = 0.32, *p* > 0.5) in that model and therefore was not added to any further models.

**Figure 3:**
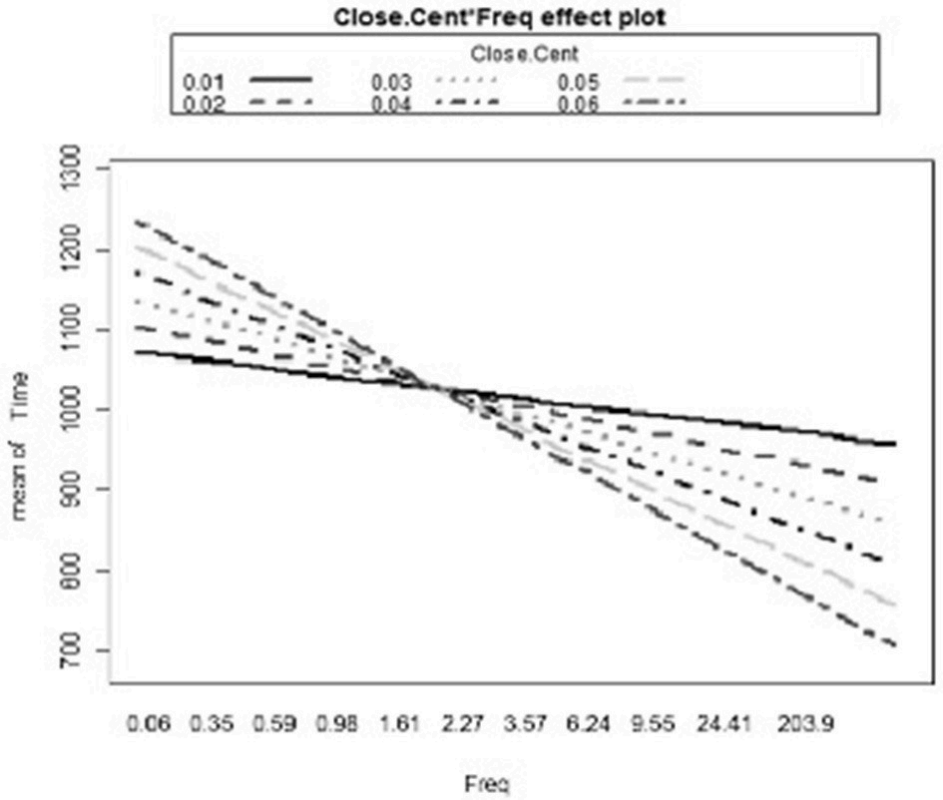
The interaction plot of the significant Frequency and Closeness Centrality interaction on reaction times.

Following the first model with reaction times as a dependent variable, a series of models was created including an interaction term in each model. A total of six different models were run, one for each interaction of a predictor (clustering coefficient, frequency of occurrence, number of neighbors, segment probability, biphone probability, and neighborhood frequency) with closeness centrality. Once again, frequency, clustering coefficient, and neighborhood frequency were significant predictors in most models (see Table 3). The only significant interaction coefficient was observed between closeness centrality and frequency (β = –3.59, *p* = 0.005). For less common words participants responded to words with high closeness centrality more slowly than to words with low closeness centrality. For more common words, participants responded to words with high closeness centrality more quickly than to words with low closeness centrality (see Figure 3).

**Table 3:** Significant predictors observed in Experiment 2 models with interaction terms and reaction time as the dependent variable.

**Interaction term included in model**	**Predictor**	**β coefficient**	***p*-value**
Closeness centrality and clustering coefficient	Frequency	−44.7	0.006
	Clustering coefficient	8.84	0.0002
	Neighborhood frequency	−0.11	0.009
Closeness centrality and frequency	Frequency	0.05	0.13
	Clustering coefficient	59.46	<0.001
	Neighborhood frequency	−0.10	0.01
Closeness centrality and number of neighbors	Frequency	−5.77	0.0002
	Clustering coefficient	24.92	<0.0001
	Neighborhood frequency	−0.09	0.01
Closeness centrality and segment probability	Frequency	−24.7	0.04
	Clustering coefficient	43.9	<0.001
	Neighborhood frequency	−0.09	0.02
Closeness centrality and biphone probability	Frequency	−79.8	0.0002
	Clustering coefficient	20.5	0.005
	Neighborhood frequency	−0.08	0.06
Closeness centrality and neighborhood frequency	Frequency	−71.98	<0.0001
	Clustering coefficient	58.41	<0.0001
	Neighborhood frequency	0.11	0.33

The same process of model creation was repeated with accuracy as the dependent variable. The model with all level 1 predictors and no interaction terms showed frequency of occurrence (β = 0.018, *p* < 0.0001) and neighborhood frequency (β = 0.00023, *p* < 0.001) as significant predictors. Once again, frequency and log mean frequency of neighborhood were significant predictors in the series of models with an interaction term included, but clustering coefficient was no longer significant in the models (see Table 4).

**Table 4:** Significant predictors observed in Experiment 2 models with interaction terms and accuracy as the dependent variable.

**Interaction term included in model**	**Predictor**	**β coefficient**	***p*-value**
Closeness centrality and clustering coefficient	Frequency	0.07	<0.0001
	Neighborhood frequency	0.0002	<0.0001
Closeness centrality and frequency	Frequency	−0.002	0.17
	Neighborhood frequency	0.0001	<0.0001
Closeness centrality and number of neighbors	Frequency	0.08	<0.0001
	Neighborhood frequency	0.0003	<0.0001
Closeness centrality and segment probability	Frequency	0.09	<0.0001
	Neighborhood frequency	0.0003	<0.0001
Closeness centrality and biphone probability	Frequency	0.06	<0.0001
	Neighborhood frequency	0.0002	<0.0001
Closeness centrality and neighborhood frequency	Frequency	0.08	<0.0001
	Neighborhood frequency	−0.001	0.07

### Discussion

The results of the present experiment show that the closeness centrality of a word interacts with the frequency with which that word occurs in the ambient language. In the case of less common words, participants responded to words with high closeness centrality (i.e., “close” to other words in the lexicon) more slowly than to words with low closeness centrality (i.e., “far” from other words in the lexicon), but in the case of more common words, participants responded to words with high closeness centrality more quickly than to words with low closeness centrality.

To account for the interaction of frequency of occurrence and closeness centrality on processing we consider the isolation effect, perhaps more commonly known as the Von Restorff effect (Von Restorff, 1933). In the Von Restorff effect unique items are remembered better than items that are similar to other items in a list. Words that occur less often in the language are, by definition, unique in the language. Furthermore, low frequency words that are “far away” from other words in the lexicon (i.e., low in closeness centrality) will be even more unique than low frequency words that are “close” to other words in the lexicon (i.e., high in closeness centrality). For example, the words DIVA and PUTTY both occur rarely in the language (DIVA occurs 1.53 per million words and PUTTY occurs 1.82 times per million words; Brysbaert and New, 2009). However, DIVA has a low closeness centrality value (0.0001) compared to PUTTY (0.065). Therefore, DIVA is not only unique due to its rare occurrence (i.e., low frequency of occurrence) in the language, but the isolation that DIVA has due to its unique location in the lexical network (i.e., low closeness centrality value) confers upon it an additional “dose” of uniqueness that leads to enhanced processing for rare words with low closeness centrality like DIVA compared to rare words with high closeness centrality like PUTTY.

Words that occur *often* in the language are, by definition, not unique. In order to become “unique,” a common word may need to rely on other characteristics in order to stand out from the other common words. As we described in Experiment 1, words with high closeness centrality might accrue benefits from being partially activated by nearby words. Words with low closeness centrality, which are far from other words in the lexicon, would not be partially activated by those “neighbors” as often, and would not be strengthened as much as words with high closeness centrality. Recall that frequency of occurrence was controlled in the stimuli used in Experiment 1, but, crucially, the frequency of occurrence of most of the words (58%) used in Experiment 1 fall above the value of 2.11, which is the approximate frequency value where words with high closeness centrality start to be responded to more quickly than words with low closeness centrality. That is, the subset of words from Experiment 2 that are comparable to the words used in Experiment 1 yield similar results, providing an important replication of the influence of closeness centrality on processing observed in Experiment 1.

The interaction found in Experiment 2 results *does* show that low frequency words are responded to more quickly when those low frequency words have low closeness centrality. This result might seem to be contradictory with other results from Experiment 2 where without interaction predictors, low frequency words were responded to more slowly than high frequency words. At first the results may seem contradictory, but recall that during the analysis where frequency of occurrence was used as a predictor, other predictor variables are held constant (including closeness centrality). In the interaction analysis, frequency of occurrence and closeness centrality are allowed to vary and it is precisely the interaction between the two variables that produced the observed results explained by the Von Restorf effect (Von Restorff, 1933).

Recall that the jTRACE lexicons were searched for words used as stimuli in Experiment 1. The same was done for words used as stimuli in Experiment 2, with only one word being found in the jTRACE lexicons. Therefore, the desired simulations comparing words with high closeness centrality to words with low closeness centrality could not be carried out.

## General discussion

In two experiments we explored how the network science measure known as closeness centrality influences processing of words in the phonological lexicon. We examined how closeness centrality may influence lexical processing in Experiments 1 and 2 by using a conventional task from Psycholinguistics, namely the auditory lexical decision task. The results of those lexical decision tasks suggest that words with high closeness centrality (i.e., words that are close to other words in the lexicon) tend to be responded to more quickly and accurately than words with low closeness centrality (i.e., words that are far from other words in the lexicon; but see Experiment 2 for an important interaction of closeness centrality with frequency of occurrence).

The results of these experiments appear at odds with several widely accepted models of spoken word recognition (McClelland and Elman, 1986; Norris, 1994; Luce and Pisoni, 1998). Recall that these models all predict that more lexical competitors will slow spoken word recognition. In the present case, these models would predict that words with high closeness centrality, or that are “close” to other words in the mental lexicon, would have more competitors and therefore be responded to slower and less accurately than words with low closeness centrality, or that are “far” from other words in the mental lexicon. However, that was not the case.

Instead, we proposed that the retrieval of a given word leads to partial activation in other related words. This partial activation can gradually strengthen a representation over time, leading to faster and more accurate retrieval of those words in the future. Words with high closeness centrality are close to other words in the lexicon, and therefore accrue much partial activation when those other words are retrieved. Words with low closeness centrality are far from other words in the lexicon, and therefore do not accrue as much benefit when those far away words are retrieved. The difference in the amount of accrued partial activation leads to the processing differences in words with high vs. low closeness centrality observed here.

Partial activation is a mechanism that is found in several widely accepted models of spoken word recognition (McClelland and Elman, 1986; Norris, 1994; Luce and Pisoni, 1998), but the effects of that partial activation accruing over time have not been widely examined in that context. However, the idea of partial activation accruing over time has been proposed in the context of Node Structure Theory (MacKay, 1982) to account for certain memory- and language-related changes that occur as we age. The idea of accrued partial activation has also been used to explain the phonological false memory phenomenon (Sommers and Lewis, 1999), and has been described as the mechanism that underlies Verbal Network Strengthening Treatment (Edmonds et al., 2009) used to treat certain types of aphasia.

The results of the present experiments, as well as of Vitevitch and Goldstein (2014), suggest that words that hold strategic positions in the lexical network (i.e., the “keywords” from Vitevitch and Goldstein (2014), or the words with high closeness centrality from the present study) can accrue partial activation when neighbors are retrieved from the lexicon. Importantly, this accrued partial activation can yield processing benefits in the future, observable even during the typically rapid process of spoken word recognition in normal, young, healthy language users. Equally important is the set of tools that network science brings to detect those strategic positions in the lexical network. Such tools are not found in more conventional approaches to studying the mental lexicon. We urge language scientists and clinicians to consider how the computational tools of network science can be used to increase our understanding of language processing and disorders (Vitevitch and Castro, 2015), and how the location of words in the lexical network—in addition to the individual characteristics of the words themselves—might also contribute to processing differences among words.

## Ethics statement

This study was carried out in accordance with the recommendations of the University of Kansas Internal Review Board with written informed consent from all subjects. All subjects gave written informed consent in accordance with the Declaration of Helsinki. The protocol was approved by the University of Kansas Internal Review Board.

## Author contributions

All authors listed have made a substantial, direct, and intellectual contribution to the work, and approved it for publication.

### Conflict of interest statement

The authors declare that the research was conducted in the absence of any commercial or financial relationships that could be construed as a potential conflict of interest.
