# Supplementary material for: The Influence of Closeness Centrality on Lexical Processing
Source: Front Psychol. 2017 Sep 26;8:1683. doi: 10.3389/fpsyg.2017.01683 (PMC5622968; doi:10.3389/fpsyg.2017.01683)
Supplement: Supplementary file 1 [file DataSheet1.docx]

**Appendix A**

Stimuli (and their characteristics) used in Experiment 1.

**Appendix B**

Stimuli (and the associated characteristics) used in Experiment 2

**Authors Note**

The experiments in this report partially fulfilled the requirements for a Doctorate degree in Psychology awarded to R.G. We thank the members of the committee (Susan Kemper, Evangelina Chrysikou, Allard Jongman and Joan Sereno) for their comments and suggestions.
